# RNA Aptamer That Specifically Binds to Mycolactone and Serves as a Diagnostic Tool for Diagnosis of Buruli Ulcer

**DOI:** 10.1371/journal.pntd.0004950

**Published:** 2016-10-24

**Authors:** Samuel A. Sakyi, Samuel Yaw Aboagye, Isaac Darko Otchere, Albert M. Liao, Thomas G. Caltagirone, Dorothy Yeboah-Manu

**Affiliations:** 1 Department of Molecular Medicine, School of Medical Sciences, Kwame Nkrumah University of Science and Technology (KNUST), Kumasi, Ghana; 2 Department of Bacteriology, Noguchi Memorial Institute for Medical Research, University of Ghana, Accra, Ghana; 3 Aptagen LLC, Jacobus, Pennsylvania, United States of America; 4 Department of Biochemistry, Cell and Molecular Biology, University of Ghana, Accra, Ghana; Fondation Raoul Follereau, FRANCE

## Abstract

**Background:**

Buruli ulcer (BU) is a subcutaneous skin disease listed among the neglected tropical diseases by the World Health Organization (WHO). Early case detection and management is very important to reduce morbidity and the accompanied characteristic disfiguring nature of BU. Since diagnosis based on clinical evidence can lead to misdiagnosis, microbiological confirmation is essential to reduce abuse of drugs; since the anti-mycobacterial drugs are also used for TB treatment. The current WHO gold standard PCR method is expensive, requires infrastructure and expertise are usually not available at the peripheral centers where BU cases are managed. Thus one of the main research agendas is to develop methods that can be applied at the point of care. In this study we selected aptamers, which are emerging novel class of detection molecules, for detecting mycolactone, the first to be conducted in a BUD endemic country.

**Methods:**

Aptamers that bind to mycolactone were isolated by the SELEX process. To measure their affinity and specificity to mycolactone, the selected aptamers were screened by means of isothermal titration calorimetry (ITC) and an enzyme-linked oligonucleotide assay (ELONA). Selected aptamers were assessed by ELONA using swab samples from forty-one suspected BU patients with *IS2404* PCR and culture as standard methods. ROC analysis was used to evaluate their accuracy and cutoff-points.

**Results:**

Five out of the nine selected aptamers bound significantly (p< 0.05) to mycolactone, of these, three were able to distinguish between mycolactone producing mycobacteria, *M*. *marinum* (CC240299, Israel) and other bacteria whilst two others also bounded significantly to *Mycobacterium smegmatis*. Their dissociation constants were in the micro-molar range. At 95% confidence interval, the ROC curve analysis among the aptamers at OD450 ranged from 0.5–0.7. Using this cut-off for the ELONA assay, the aptamers had 100% specificity and sensitivity between 0.0% and 50.0%. The most promising aptamer, Apt-3683 showed a discernible cleavage difference relative to the non-specific autocatalysis over a 3-minute time course.

**Conclusion:**

This preliminary proof-of-concept indicates that diagnosis of BUD with RNA aptamers is feasible and can be used as point of care upon incorporation into a diagnostic platform.

## Author Summary

Buruli ulcer disease is a subcutaneous necrotizing skin disease caused by ecological *Mycobacterium ulcerans*. The availability of antibiotic treatment makes it imperative for early detection of the disease to avoid the disfiguring and morbid effects associated with the disease. Currently available detection methods include: microscopy for detecting acid-fast bacilli, culture to isolate viable organism, and PCR for detecting pathogen specific DNA which is usually *IS2404* and histopathology. However, most of these methods have major drawbacks, they are done in referenced laboratories and cannot be used in the field where the disease is most prevalent. *M*. *ulcerans* produces a lipid chemical called mycolactone, which is accountable for the virulent nature of the disease and has been postulated as a diagnostic target. The lipid nature of the mycolactone however makes it difficult for the body to produce antibodies against it. We therefore designed nucleic acid detection molecules that have high affinity to its target called aptamers. This aptamers were selected to detect mycolactone and hence *M*. *ulcerans*. The selected aptamers were tested with clinical samples and concluded preliminarily that, aptamers can be raised against mycolactone and this be used as point of care upon optimization and subsequent incorporation into a diagnostic platform.

## Introduction

Buruli ulcer Disease (BUD) has been listed among neglected tropical diseases, the causative agent is an ecological pathogen known as *M*. *ulcerans* [1,2]. Among mycobacterial diseases, it is the third utmost after tuberculosis and leprosy. It has been recounted in more than 30 tropical countries. The main problem however, is concentrated in West Africa where it has assumed the second most imperative mycobacterioses [3]. It is characterized by widespread debilitation of soft tissues and skin with the development of huge ulcers typically located on body extremities [4,5]. Although mortality is low, indisposition and resulting functional disability can be severe [6–9]. As a result, the societal and financial burden of BUD can also be high, especially in poor rural areas.

The mode of pathogen transmission and host immune response to infection is not fully understood; hence current control strategy is centered primarily on early identification of cases, antimycobacterial administration and wound management. The present World Health Organization (WHO) treatment includes everyday administration of oral and intramuscular streptomycin and rifampicin respectively for eight weeks. Surgical removal of foreign materials and dead tissues from progressing wounds and/or skin grafting, may be required to prevent secondary infections, enhance healing, and to rectify disfigurements. [10]. The administration of antimycobacterial drugs has made laboratory validation of clinically assumed cases very critical for treatment of BU. Even though the overall observation is that diagnosis centered on clinical decision only is satisfactory, instances of wrong diagnosis have been described [11–13]. Due to cost, infrastructural and expertise demand, the current WHO recommended gold standard diagnostic protocol (*IS2404* detecting PCR) has rendered bacteriological validation to a quality control means for diagnosing BUD. There is therefore the need to research into development of simpler methods that can be applied at the point of care.

A distinguishing feature of *M*. *ulcerans* amongst human mycobacteria, is the secretion of mycolactone [14], the virulent factor responsible for the pathogenesis of the disease. Intact mycolactone has been found to be present in biological materials collected from all forms and stage of BUD [15]. Furthermore, there is proof from mouse and human experimentation that mycolactone is detectable in peripheral blood [16] and has been postulated as a useful marker for diagnosis. However, the chemical nature of mycolactone as a poor immunogenic lipid molecule has impeded efforts to produce an immunodiagnostic based detection of mycolactone.

An aptamer is a nucleic acid molecule (single-stranded DNA or RNA) that binds to its target with high specificity and affinity [17,18]. Aptamers do not carry genetic information but work through affinity binding to their target [19]. They interact with their targets via secondary and/or tertiary structures [20]. Aptamer, upon binding to its target via the binding domain, allosterically transfers stability to other component of the structure. Aptamers are raised *in vitro* by a PCR-based, iterative procedure called systemic evolution of ligands by exponential enrichment (SELEX). Likened to protein antibodies, aptamers have several benefits like quick synthesis, high affinity, low-temperature sensitivity, can be manufactured on a large scale and can be easily adjusted biochemically [21,22]. Aptamers have been applied in many areas such as biosensor, medical diagnosis and as a therapeutic tool [23,24]. To date, this novel, profound and explicit class of recognition molecules, has previously not been explored for BU diagnosis. In this proof of concept study we explored the feasibility of raising aptamers against mycolactone as a simpler diagnostic tool for BUD.

## Methods

### Ethics Statement

The protocol for this study was reviewed and ethical approval granted by the Institutional Review Committee of the Noguchi Memorial Institute for Medical Research (NMIMR), of the University of Ghana. Consent was sought from grown-up participants and legal guardians of all minors.

### Extraction of mycolactone

A clinical *M*. *ulcerans* strain (NM209) was used as an in-house reference. This strain was isolated from skin lesion of a female patient in December, 2009. This strain was sub-cultured into Middlebrook 7H9 broth enriched with 10% OADC (Sigma-Aldrich, MO, USA) and incubated at 32°C. The third week culture was further sub-cultured into six more Middlebrook 7H9 broth for four weeks into mid-log phase with intermittent shaking. The bacteria cells were harvested, dried, and weighed. Chloroform-methanol (2:1, vol, /vol) and a magnetic stirrer were used to stripped lipids from the cell wall at 4°C for 24hrs [25]. A rota-evaporator was then used to dry the organic phase after which it was re-suspended in ice-cold acetone. This was then incubated for 20hrs at -20°C after which it was loaded on a TLC silica gel plate with chloroform, methanol and water as mobile phase (90:9:1). A yellow band, which is indicative of mycolactone was scraped and eluted from the silica using 2/1 chloroform, methanol (v/v) [26]. Synthetic mycolactone was used as control. Purified mycolactone was re-suspended in ethanol after solvent evaporation. UV absorption was used to determine the concentration of the resulting solution [27].

### Selection of RNA aptamers against mycolactone

The initial aptamer library template and primers were synthesized by IDT (Coralville, IA) as single-stranded DNA. The library was then primer extended to provide double-stranded DNA (dsDNA) using Titanium Taq DNA polymerase from Clontech (Mountain View, CA). The extracted mycolactone and bacterial cell protein extract were used as target and counter- target respectively. For a given generation of the library, RNA was transcribed from the previous dsDNA with AmpliScribe T7 Transcription kits from Epicentre (Madison, WI) and purified using a 10% denaturing polyacrylamide gel electrophoresis (PAGE). The purified RNA was combined with selection buffer, which was then diluted to 1X concentration (1X PBS [pH 7.4] and 10 mM MgCl_2_) for negative selection. After incubation, non-cleaved RNA was separated from cleaved RNA using 10% denaturing PAGE. Recovered non-cleaved material was combined with counter-target and buffer, target and buffer, or buffer alone depending on the selection step, incubated, and partitioned on 10% denaturing PAGE. Recovery and another selection step was implemented. cDNA was then generated from eluted post-selection library using SuperScript II Reverse Transcriptase (Life Technologies; Carlsbad, CA), then PCR-amplified with Titanium Taq DNA polymerase (Clontech; Mountain View, CA) to complete the round of selection. Selection steps for a particular round varied depending on what condition needed to be improved based on results from the prior round. After selections were completed, cDNA of the resultant enriched library was PCR amplified, transcribed, and then divided into three samples, with one exposed to the selection buffer alone, one exposed to the counter-target protein in selection buffer, and the last exposed to the target (mycolactone) in selection buffer. Material that cleaved after binding to elements of its treatment condition was recovered, reverse transcribed, PCR amplified and sequenced.

### Sequence analysis and aptamer candidate selection

The Illumina MiSeq system (San Diego, CA) was employed to sequence the parallel library product after the selections to generate single-end reads. Bioinformatics analysis of the sequencing data identified candidate aptamer molecules. The deep sequencing and subsequent data analysis reduced the traditional approach of performing a large number of selections, which may introduce error and bias due to the screening process [28]. Sequence family construction focused on motif presence. Three libraries were collected from the parallel assessment: the positive target-exposed library, the buffer-only negative library, and the counter-target-exposed library. All libraries were analyzed to discover any sequences that have yet to be removed during a negative- or counter-selection step, but still have affinity for both the target and counter-target. Sequence families with lower numbers but a higher representation in the positive population were identified as potential candidates [29].

### Estimation of constant of dissociations (Kd) of selected aptamers

To estimate the affinity of the RNA aptamers to mycolactone, kinetic analysis were done with isothermal titration calorimetry (VP-ITC), (MicroCal, LLC, MA). The first injection was disregarded. The models independent and blank (constant) were used for all runs. Running conditions consisted of 33 injections of 3ul at 25°C with 70ul of aptamer and 300ul of mycolactone. Control titrations (mycolactone titrated into buffer) were subtracted from the data before fitting. The integration width used in all cases was 180s. The evaluation of the data was done using NanoAnalyze software version 3.1

### Assessment of binding ability of RNA aptamers to mycolactone by ELONA

Specific aptamers selected by proprietary bioinformatics were checked for their binding affinity to mycolactone using ELONA assay [18,30]. Briefly, each RNA aptamer was biotinylated by IDT (Coralville, IA). Fifty microliters (50ul) of mycolactone in 10mls of NaCO_3_ buffer were aliquoted into a 96-well microtiter plates at 4°C and left overnight. A 5% fat-free milk was used to block the solution at 4°C for 1 hour, TBS buffer was then used to wash the wells four times. 500 nM of biotinylated aptamers were aliquoted into each well and incubated at room temperature for 2 hours. Subsequently, the wells were washed again with TBS buffer. Streptavidin-horseradish peroxidase conjugate (KPL) was diluted 1:15 000 in TBS buffer, and 100ul was aliquoted to each well. The plates were incubated at 37°C for 2 hours and washed as previously described. Next, 50ul of Turbo-3, 3’, 5, 5’-tetramethylbenzidine (TMB, Pierce) was aliquoted into individual wells and incubated at 37°C for 15 minutes. The reaction was stopped by the addition of 50ul of 1M H_2_SO_4_ and the resulting complexes were measured at absorbance 450 nm using a MultiSkan Go plate reader (Thermo Scientific).

### Specificity testing of aptamers

To evaluate their specificity, all the five RNA aptamers were assessed in an ELONA assay to determine their ability to distinguish among several bacterial lysate mostly associated with wounds and ulcers. The bacteria include: *Mycobacterium bovis BCG*, *Staphylococcus aureus*, *Pseudomonas aeruginosa*, *Proteus mirabilis*, *Mycobacterium smegmatis*, *Mycobacterium tuberculosis*, *Mycobacterium ulcerans*, *Mycobacterium marinum* (CC240299, Israel) *TB H3Ra*, *Enterobacter*, *Klebsiella pneumonia and Escherichia coli*. Two hundred microliters (200ul) of bead-beaten lysates from each bacteria culture was used. Equal amount of these bacterial lysate were aliquoted into microtitre plates using *M*. *ulcerans* as the positive control.

### Detection of mycolactone in clinical samples

To evaluate the use of the selected RNA aptamers as a recognition technique, the aptamers were tested in a case-controlled study. Swabs and FNA specimen were taken from forty-one BU suspected patients from three districts in the West and South districts of the Greater Accra region. Specimens were collected using WHO prescribed standard procedures [2]. Swab samples were taken from ulcerative lesions by swabbing the surface underneath the undermined edges. In patients with pre-ulcerative lesions, FNA specimen was taken from the core of the lesion [31]. The collected swabs were put into 15mL falcon tubes with transport medium that covers the whole tip of the swab. On the other hand, FNA samples were drained into an Eppendorf tube containing 500μL phosphate buffered saline (PBS). For processing and analysis, specimens were sent to the Noguchi Memorial Institute for Medical Research (NMIMR). The Institutional Review Committee of the NMIMR provided the ethical approval for the study. Consent was sought from grown-up participants and legal guardians of all minors. The samples were analyzed by PCR for the *IS2404* sequence repeat, culture, and ELONA assay. An ELONA was done to determine if the aptamers will be able to identify mycolactone present in the samples. Culture and *IS2404* PCR results from the same samples were used as standard. Specificity and sensitivity was compared between the ELONA assay, *IS2404* PCR and culture results.

### Specific aptamer cleavage testing

The most specific aptamer Apt-3683 was tested for its ability to detect differences in percentage cleavage in the presence and absence of mycolactone. Specific Apta-3683 (0.5mM) mediated by the presence of mycolactone (100 mM) over a 3 minute time course was done. The cleavage percentages were generated by running sample against 20/100 DNA Ladder from IDT (Coralville, IA), stained with Gel Star (Lonza; Basel, CH) and photo-documented under UV trans-illumination. Reaction conditions, volumes and the subsequent PAGE analysis also included the additional time points of 120 seconds and 180 seconds.

### Statistical analysis

For all the ELONA binding tests, an aptamer alone control was used for each plate. The average was taken and subtracted from the individual well to get rid of background noise. Individual aptamers or aptamer-mycolactone/bacterial lysate mixture were tested in duplicate. This was also averaged and used to calculate the standard deviation. NanoAnalyze software was used to calculate for the dissociation constant. The ROC curve analysis was used to determine the diagnostic accuracy of the aptamer based test (GraphPad Prism 5.04, GraphPad Software, Inc.)

## Results

### Selection of RNA aptamers

**Fig 1:**
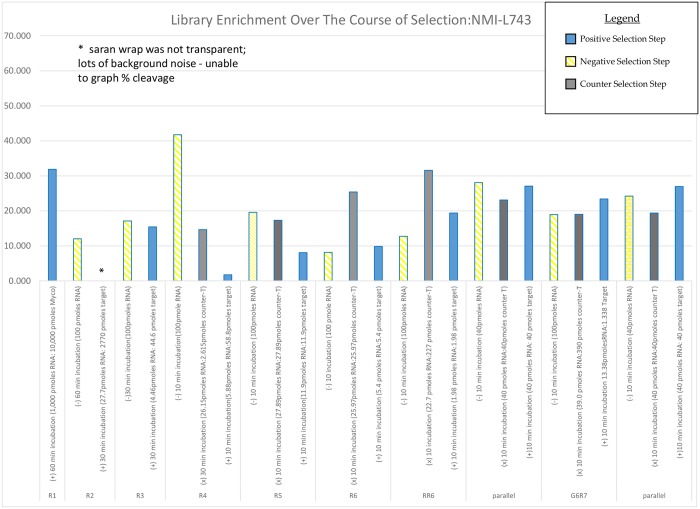
Progress of library enrichment over the course of selection. Gel images taken after partitioning step to separate responsive from non-responsive sequences were analyzed using the “Analyze -> Gels” functions from Image J (NIH; Bethesda, MD). Densitometry values were then processed to yield a ratio of cleaved: total material as a percentage. Cleaved material from the last “Parallel” was isolated for sequencing and analysis.

Mycolactone (0.2mg/ml) in 0.5 ml ethanol was obtained of which 1000pmoles were used as target in the aptamer selection process. After seven rounds of selection cycle, the library responded to the target at a higher rate than the counter-target or buffer-only conditions. Sequence family construction focused on motif presence. In all, 197007 sequences were analyzed from the positive target-exposed library. From this set of data, 6859 sequence families were constructed containing between 10 and 583 members each. The M-fold Zuker algorithm dependent program was used to predict the secondary structure of a given candidate [32] to ensure that the aptamer can structurally shift to an active form. Fig 1 shows library enrichment over the course of SELEX process indicating percentage cleavage for positive, negative and counter-selections.

### Binding of RNA aptamers to mycolactone

**Fig 2:**
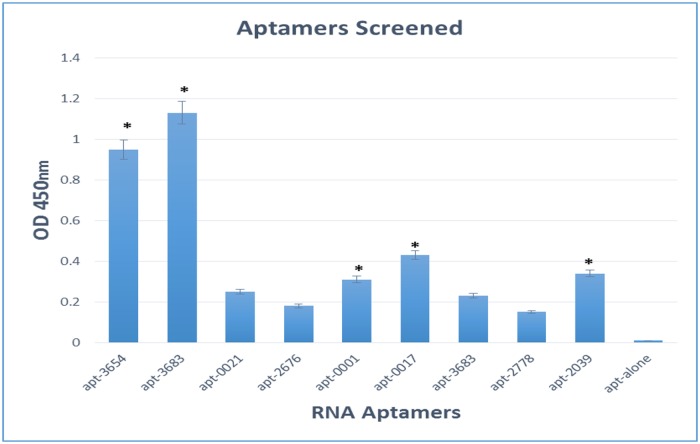
Binding affinity of RNA aptamers to mycolactone. Biotinylated RNA aptamers that bound significantly to mycolactone in an ELONA assay are denoted by the asterisks.

Nine RNA aptamers were selected, of these five aptamers designated APT-3659, APT-0017, APT-2039, APT-3683 and APT-0001 were identified to be specific to mycolactone. Out of the nine RNA aptamers screened against mycolactone, five bound significantly (p, 0.0035) displaying high OD450 readings and were selected for additional analysis. Fig 2 show how the various isolated RNA aptamers bound to mycolactone.

**Fig 3:**
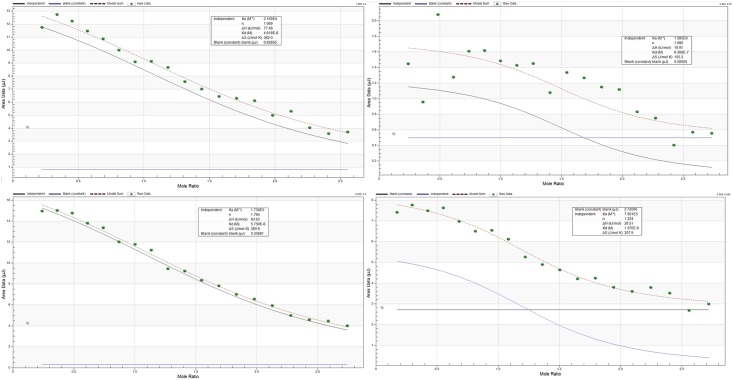
ITC thermogram showing the equilibrium dissociation constants (KD) of the selected RNA aptamers. Panel A: thermogram of apt-3683, Panel B: thermogram of apt-3654, Panel C: thermogram of apt-2039, Panel D: thermogram of apt-0017. In each case the injection volume was 3ul, injection rate was 0.5ul/seconds with 180 seconds spacing.

To evaluate the binding kinetics of selected aptamers, isothermal titration calorimetry method was used to obtain the constant of dissociation (kd) values for each aptamer. The aptamers gave kd results in the lower micro-molar range from 1.59–73.0 uM. Fig 3 depicts isothermal titration calorimetry thermogram of the aptamers. They all gave a sigmoidal curve indicative of strong affinity of the aptamers to mycolactone.

### Sensitivity and specificity of the selected aptamers

The specificity of the RNA aptamers were tested by lysates prepared from bacteria mostly found inhabiting wounds and ulcers and were checked for their ability to bind to aptamers. The five selected aptamers had varied binding abilities even though all aptamers were able to recognize *M*. *ulcerans* lysates, two aptamers, however, bound to the lysates of *M*. *smegmatis*. Table 1 shows ELONA results of RNA aptamers tested on various bacterial lysates. (Table 1). Additionally, for sensitivity testing, decreasing concentrations of the most promising aptamer (0–100mM) was used to determine the least concentration at which the aptamer would cleave in the presence of mycolactone **(1μM)** as shown in supplementary 1 (S1 Fig).

**Table 1:** ELONA results on RNA aptamers tested on different bacterial lysates.

Bacterial Lysate	Apt-3654	Apt-3683	Apt-0017	Apt-3683	Apt-0001	Apt-2039
***Mycobacterium ulcerans***	**+**	**+**	**+**	**+**	**+**	**+**
***TB H3Ra***	**-**	**-**	**-**	**-**	**-**	**-**
***Klebsiella pneumoniae***	**-**	**-**	**-**	**-**	**-**	**-**
***M*. *marinum*** (CC240299, Israel)	**-**	**-**	**-**	**-**	**-**	**-**
***E*. *Coli***	**-**	**-**	**-**	**-**	**-**	**-**
***Staph aureus***	**-**	**-**	**-**	**-**	**-**	**-**
***Pseudomonas aeruginosa***	**-**	**-**	**-**	**-**	**-**	**-**
***Enterobacter***	**-**	**-**	**-**	**-**	**-**	**-**
***Proteus mirabilis***	**-**	**-**	**-**	**-**	**-**	**-**
***M*. *smegmatis***	**-**	**-**	**-**	**-**	**+**	**+**
***TB bovis***	**-**	**-**	**-**	**-**	**-**	**-**
***TB BCG***	**-**	**-**	**-**	**-**	**-**	**-**
***TB H37RV***	**-**	**-**	**-**	**-**	**-**	**-**

### Mycolactone detection from clinical swabs samples

To assess the aptamers capacity to detect *M*. *ulcerans* infection using clinical swab samples, the aptamers were tested in forty-one clinical swab samples in an ELONA assay with culture and *IS2404* PCR as standard methods. Fourteen swab samples tested positive for both culture and *IS2404* PCR whilst positivity observed among the aptamers ranged from one to seven (Table 2). ROC curve analysis with a 95% confidence interval observed among the aptamers at OD450 ranged from 0.5–0.7. Using this cut-off for the ELONA assay, the aptamers had 100% specificity and sensitivity between 0.0% and 50.0%. Table 2 shows the aptamer’s ability to detect *M*. *ulcerans* infection using clinical swab samples in an ELONA assay with culture and *IS2404* PCR as standard methods, while Table 3 indicate ELONA results of five RNA aptamers tested as a detection reagent for BU from clinical samples with culture and *IS2404* PCR as standard.

**Table 2:** Aptamers ability to detect *M*. *ulcerans* infection using clinical swab samples in an ELONA assay with culture and *IS2404* PCR as standard methods.

		Aptamers				
*IS2404* PCR/CULTURE	Total	apt-3683	apt-3654	apt-2039	apt-0017	apt-0001
**Sample size**	41	41	41	41	41	41
**Positives:**	14	7	4	2	1	0
**Negatives:**	27	34	37	40	40	41

**Table 3:** ELONA results of five RNA aptamers tested as a detection reagent for BU from clinical samples with culture and *IS2404* PCR as standard.

Aptamers	AUC (95% CI)	Sensitivity (95% CI)	Specificity (95% CI)	PPV	NPV	p-value
**Apt-3683**	0.8 (0.6–0.9)	50.0% (23.0–77.0)	100%(87.2–100.0)	100.0%	79.0%	**0.0003**
**Apt-3654**	0.6 (0.5–0.8)	28.6% (8.4–58.1)	100%(87.2–100.0)	100.0%	73.0%	**0.0226**
**Apt-2039**	0.5 (0.4–0.7)	14.3% (4.2–29.1)	100%(87.2–100.0)	100.0%	67.5%	0.3173
**Apt-0017**	0.5 (0.4–0.7)	7.1% (0.2–33.9)	100%(87.2–100.0)	100.0%	67.5%	0.3173
**Apt-0001**	0.5 (0.3–0.7)	0.0% (0.0–23.2)	100%(87.2–100.0)	100.0%	65.9%	1.0000

AUC: area under the curve; CI: confidence interval; PPV: positive predicted value; NPV: negative predicted value

### Specific aptamer cleavage percentage testing

**Fig 4:**
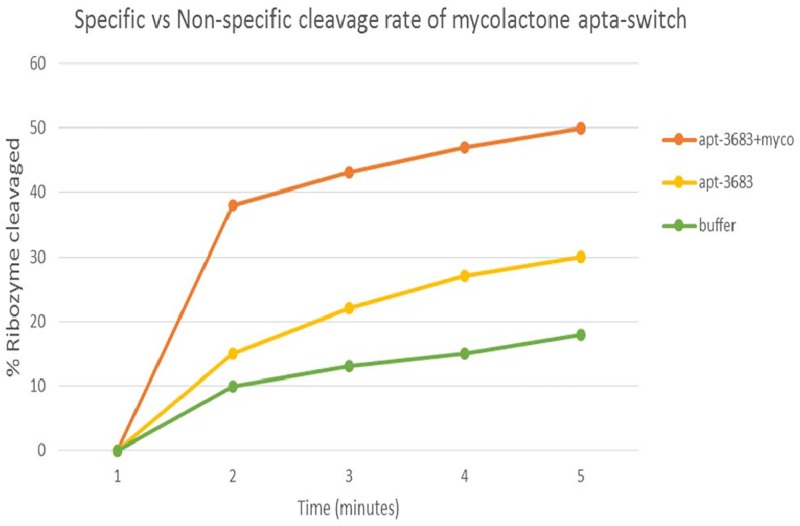
Specific verses non-specific cleavage rate of apt-3683 in the presence & absence of mycolactone.

Fig 4 shows the most significant and specific aptamer Apt-3683 showing a discernible cleavage difference relative to the non-specific auto-catalysis that occurs in the presence and absence of mycolactone or buffer alone over time a 3 minute time course.

## Discussion

The current study used the allosteric ribozyme design as a template to select aptamers. The aptamers were selected with mycolactone as a target to be used as a potential *M*. *ulcerans* detection assay. In all, nine individual RNA aptamer candidates were isolated from which five bound significantly to mycolactone. Further characterizations of the aptamers indicate aptamers had a high affinity to mycolactone, moreover, the selected aptamers had constants of dissociation in the lower micro-molar range. The selected aptamers were further shown to discriminate between mycolactone producing mycobacteria, *M*. *marinum* (CC240299, Israel) and other gram positive and gram negative bacteria normally present in wounds and ulcers [33]. The binding ability of the aptamers were tested against the various bacterial lysates and it was observed that the aptamers were able to discern among most of the bacteria and mycolactone producing *M*. *ulcerans*. However, Apt-0017 and apt-0001, also bound significantly to *M*. *smegmatis*. This may be due to the fact that aptamers bind preferentially to functional parts of proteins, and thus a functional protein within the binding domain of *M*. *smegmatis* may be interacting with the two aptamers. It is interesting to note that these two aptamers also bound significantly to *M*. *ulcerans*. The aptamer-based assay was used in a case control study and had a sensitivity of 50% and a specificity of 100%. This is promising for development of the aptamers as recognition molecules for diagnosing BU. The aptamer-based test had a sensitivity of 50% which is comparable to that of microscopy and culture [6,34] whilst the specificity is comparable to that of the *IS2404* PCR [35,36]. In the current study, it was further observed that the aptamer-based detection assay did reasonably well in patients with active BUD. Compared to routine diagnostic tests, aptamer-based detection assay have better stability under a varied circumstances, and can be used repetitively. They can thus, serve as detection molecules for the development of better diagnostic assays [37]. Furthermore, the current study and other experimentations have revealed that aptamers can be synthesized chemically, this will allow for the aptamers to be manufactured rapidly on a large scale [37].

A limitation is the absence of a speedy readout, as it takes many hours to finish an ELONA, in addition to antigenic cross-reactivity. The optimization of this aptamer-based detection assay will improve its sensitivity and reduce the effect of cross-reactivity, this can be done by truncation of the present 88-mer mother aptamers to just the necessary sequences needed for binding to the target, and this can also improve the kinetics of the aptamers [38]. This will further reduce cost of synthesizing the aptamers and prevent non-specific binding. Furthermore, changing the communication module with renowned modules like flavin mononucleotide (FMN) and theophylline communications modules can improve the sensitivity of the assay. The current study has thus revealed that it is practical to select specific aptamers to *M*. *ulcerans* target and identify this target in patients with active form of the disease. However, further optimization is required to improve the sensitivity and hence performance outcomes, followed by justification in bigger a study using clinical specimen at different stage of the disease. This will form the basis for aptamers to be included in point-of-care diagnostic platforms.

## Supporting Information

S1 Fig Decreasing aptamer concentrations (0–100 mM) reactions carried out in the presence of mycolactone (1μM). Results show a discernable difference down to 10 mM.
(TIF)
