# Supplementary figures and images for: RNA Aptamer That Specifically Binds to Mycolactone and Serves as a Diagnostic Tool for Diagnosis of Buruli Ulcer
Source: PLoS Negl Trop Dis. 2016 Oct 24;10(10):e0004950. doi: 10.1371/journal.pntd.0004950 (PMC5077154; doi:10.1371/journal.pntd.0004950)

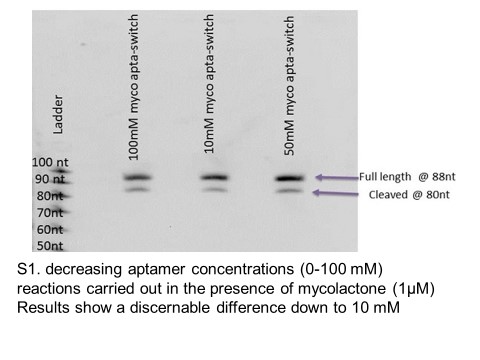

Supplement: S1 Fig — Results show a discernable difference down to 10 mM. (TIF) [file pntd.0004950.s001.tif]
